# Predictive Symptoms and Signs of Severe Dengue Disease for Patients with Dengue Fever: A Meta-Analysis

**DOI:** 10.1155/2014/359308

**Published:** 2014-07-01

**Authors:** H. Zhang, Y. P. Zhou, H. J. Peng, X. H. Zhang, F. Y. Zhou, Z. H. Liu, X. G. Chen

**Affiliations:** ^1^Department of Infectious Diseases and Hepatology Unit, Nanfang Hospital, Southern Medical University, Guangzhou 510515, China; ^2^Key Laboratory of Prevention and Control for Emerging Infectious Diseases of Guangdong Higher Institutes, School of Public Health and Tropical Medicine, Southern Medical University, Guangzhou 510515, China; ^3^Department of Infectious Diseases, The Third Affiliated Hospital of Sun Yat-sen University, Guangzhou 510630, China

## Abstract

The aim of the meta-analysis was to provide more solid evidence for the reliability of the new classification. A systematic literature search was performed using PubMed, Armed Forces Pest Management Board Literature Retrieval System, and Google Scholar up to August 2012. A pooled odds ratio (OR) was calculated using either a random-effect or a fixed-effect model. A total of 16 papers were identified. Among the 11 factors studied, five symptoms demonstrated an increased risk for SDD, including bleeding [OR: 13.617; 95% confidence interval (CI): 3.281, 56.508], vomiting/nausea (OR: 1.692; 95% CI: 1.256, 2.280), abdominal pain (OR: 2.278; 95% CI: 1.631, 3.182), skin rashes (OR: 2.031; 95% CI: 1.269, 3.250), and hepatomegaly (OR: 4.751; 95% CI: 1.769, 12.570). Among the four bleeding-related symptoms including hematemesis, melena, gum bleeding, and epistaxis, only hematemesis (OR: 6.174; 95% CI: 2.66, 14.334; *P* < 0.001) and melena (OR: 10.351; 95% CI: 3.065, 34.956; *P* < 0.001) were significantly associated with SDD. No significant associations with SDD were found for gender, lethargy, retroorbital pain, diarrhea, or tourniquet test, whereas headache appeared protective (OR: 0.555; 95% CI: 0.455, 0.676). The meta-analysis suggests that bleeding (hematemesis/melena), vomiting/nausea, abdominal pain, skin rashes, and hepatomegaly may predict the development of SDD in patients with DF, while headache may predict otherwise.

## 1. Introduction

Dengue is an infectious disease caused by dengue virus (DENV). It is endemic in many tropical and subtropical areas. Patients infected with DENV have a wide spectrum of clinical manifestation, ranging from silent infections with no symptoms to a mild flu-like syndrome, dengue fever (DF), or severe dengue disease (SDD), including dengue haemorrhagic fever (DHF) and dengue shock syndrome (DSS) [1–3]. Recently, DF has become one of the most challenging public health problems in affected regions, as the DF incidence increases rapidly worldwide [4]. There are approximately 2.5 billion people at risk for DF worldwide. Fifty million people would acquire DENV annually, and half a million among them would develop dengue hemorrhagic fever, including 22,000 deaths [5].

Several methods have been used for the diagnoses of DF. However, there lacks an accurate means to predict the severity of disease at early stages of the infection. Since patients with mild or classical DF can develop SDD later [2], it is important to look for symptoms/signs to facilitate the early prediction of the progression into SDD. The establishment of predictive symptoms/signs is essential for preventing unnecessary hospitalization, reducing disease burden, and controlling potential SDD. Based on the dengue guidelines (2009), the warning symptoms/signs for SDD include abdominal pain or tenderness, persistent vomiting, mucosal bleed, lethargy, and restlessness.

Published studies about symptoms/signs that are associated with SDD have been inconclusive. For instance, Khan et al. found that male DF patients were more likely to progress into DHF (OR: 2.3, 95% CI: 1.1–4.5, *P* value 0.021) [6], while there was no association with SDD [7, 8]. The frequencies of symptoms/signs of vomiting/nausea, abdominal pain, skin rashes, and bleeding were also found to be correlated with SDD [6–11]. However, the published studies were not able to conclude that these symptoms/signs are associated with SDD. In addition, although some findings such as viral factors, varying host immune conditions, host immune reactions, and laboratory tests can predict SDD [12], the clinical manifestations might always offer the earliest markers in predicting SDD. For example, patients with nonsevere dengue could be clustered into two groups: one with warning signs, such as abdominal pain, mucosal bleeding, and liver enlargement, and the other without those signs [2], as most of the warning signs were associated with an indication for ICU admission and were severe, even for the relationship of death [13].

Because of these inconsistent reports, more accurate methods to predict SDD are needed. We conducted the meta-analysis to identify which clinical symptoms/signs are associated with SDD and to help find better methods to predict the development of SDD in patients with DF.

## 2. Materials and Methods

### 2.1. Literature Searches

Our study was performed according to the recommendations of the PRISMA Statement [14], which is available in supporting information (see Table S1 available online at http://dx.doi.org/10.1155/2014/359308). Computerized searches were conducted on NCBI PubMed, Armed Forces Pest Management Board Literature Retrieval System, and Google Scholar. As few studies before 2000 met the criteria of WHO guidelines (1997), the search time window was set between January 1, 2000, and August 1, 2012, with no language limit. Because severe dengue disease (SDD) is classified as DHF and DSS, we used the following key words for searching: dengue fever, DF, dengue haemorrhagic fever, DHF, dengue shock syndrome, DSS, and clinical diagnosis. We also manually searched the reference lists of the retrieved articles to identify more qualified studies.

### 2.2. Inclusion and Exclusion Criteria

Studies were eligible for inclusion if they met the following criteria: (1) retrospective, prospective, or cross-sectional studies providing the details of symptoms/signs as well as any information regarding gender, vomiting/nausea, abdominal pain, skin rashes, bleeding, headache, lethargy, retroorbital pain, diarrhea, hepatomegaly, or tourniquet test; (2) the symptoms/signs of DF and SDD were distinguished; (3) cases with DF in the study were confirmed by laboratory tests; cases with SDD were defined by one or more of the following: plasma leakage that may lead to shock (dengue shock) and/or fluid accumulation, with or without respiratory distress, and/or severe bleeding, and/or severe organ impairment. When two or more publications reported the same study, we chose the most recent one. Reports providing inadequate information were excluded.

### 2.3. Quality Assessment

The quality of the selected studies was assessed independently by two authors using the Newcastle-Ottawa Scale (NOS) [15]. The NOS uses different tools for case-control and cohort studies and consists of 3 parameters of quality: selection, comparability, and exposure/outcome assessment. The NOS assigns a maximum of 4 points for selection, 2 for comparability, and 3 for exposure or outcome. We assigned NOS scores of 1–3, 4–6, and 7–9 for low, intermediate, and high-quality studies, respectively. Discrepancies were settled by consensus after joint reevaluation of the original studies.

### 2.4. Data Extraction

For each eligible manuscript, the following information was extracted: (1) first author's name and year of publication; (2) study design (prospective, retrospective, or cross-sectional); (3) study populations (children, adults, or both); (4) distinctive numbers of patients with specific symptoms in DF and SDD groups.

### 2.5. Statistical Analysis

The prevalence rates of specific symptoms/signs in DF and SDD groups were compared by calculating an odds ratio (OR) with a 95% confidence interval (CI) using either a fixed-effect model or a random-effect model. Predictive factors of interest included gender, vomiting/nausea, abdominal pain, skin rashes, bleeding (hematemesis, melena, gum bleeding, and epistaxis), headache, lethargy, retroorbital pain, diarrhea, hepatomegaly, and tourniquet test.

Heterogeneity between studies was assessed using both the Chi-square test with a *P*  value ≤0.10 and the inconsistency index (*I* ^2^) with a cut-off of 50% [16]. To explore the potential sources of heterogeneity among studies, subgroup analyses and metaregression were performed on the strata of study design, study population, and publication year.

Potential publication bias was comprehensively assessed by Begg's funnel plot and Egger's rank correlation test of asymmetry. Publication bias was determined present when the *P* value ≤0.10 by Egger's or Begg's test. All statistical analyses were performed using STATA version 11.0 (STATA Corporation, College Station, TX, USA).

## 3. Results

### 3.1. Study Characteristics and Quality

**Figure 1:**
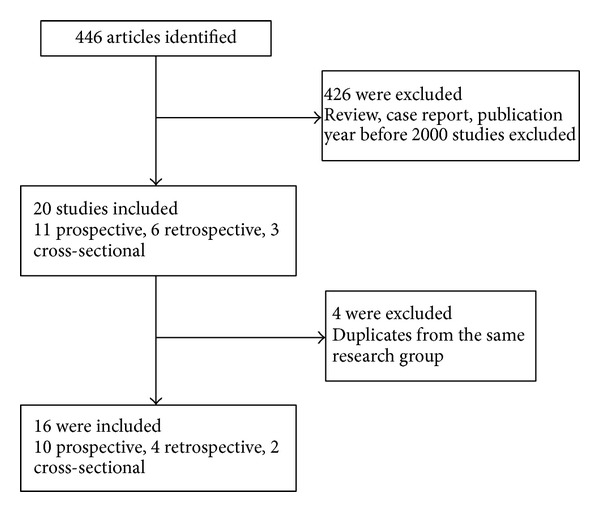
Flow diagram of selection and disposition of studies.

The search strategy identified 446 citations. Sixteen articles published between 2000 and 2012 were ultimately included in this meta-analysis based on the inclusion and exclusion criteria (Figure 1). The final collection consists of 10 prospective [9–11, 17–23], four retrospective [7, 8, 24, 25], and two cross-sectional studies [26, 27]. As listed in Table 1, eight of the 16 studies reported on a population study of children, two on adults and six on both. The factor of gender was included in five studies. The clinical symptoms/signs of vomiting/nausea were included in 13 studies, abdominal pain in 13, skin rashes in 10, bleeding in 13, headache in 13, lethargy in 6, retroorbital pain in 9, diarrhea in 7, hepatomegaly in 8, and tourniquet test in 4 studies. Four common kinds of bleeding symptoms were present in these studies, including hematemesis in five, melena in four, gum bleeding in seven, and epistaxis in five. Based on the NOS scores, 12 studies (75%) were of high quality and the other four (25%) were acceptable.

### 3.2. Potential Predictive Indicators of SDD

**Figure 2:**
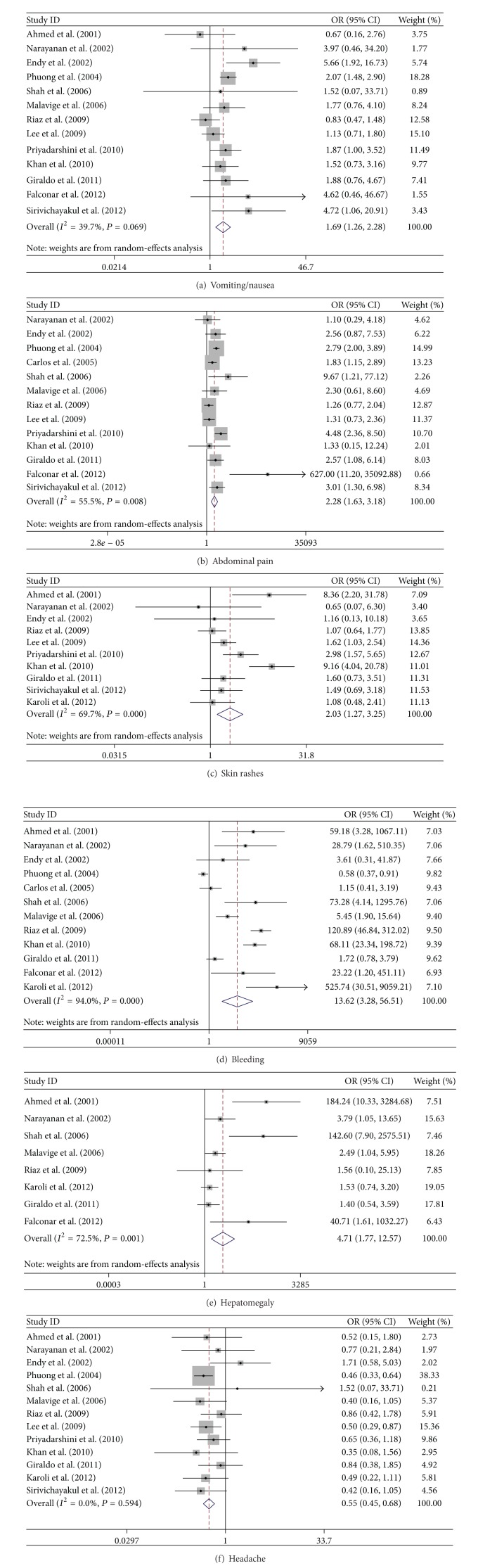
Forrest plots of the relationship between DF and the risk of SDD. (a)–(e) Pooled ORs of SDD are greater than one in 5 symptoms and signs with vomiting/nausea, abdominal pain, skin rashes, bleeding, and hepatomegaly; (f) pooled OR of SDD is smaller than one in headache.

**Figure 3:**
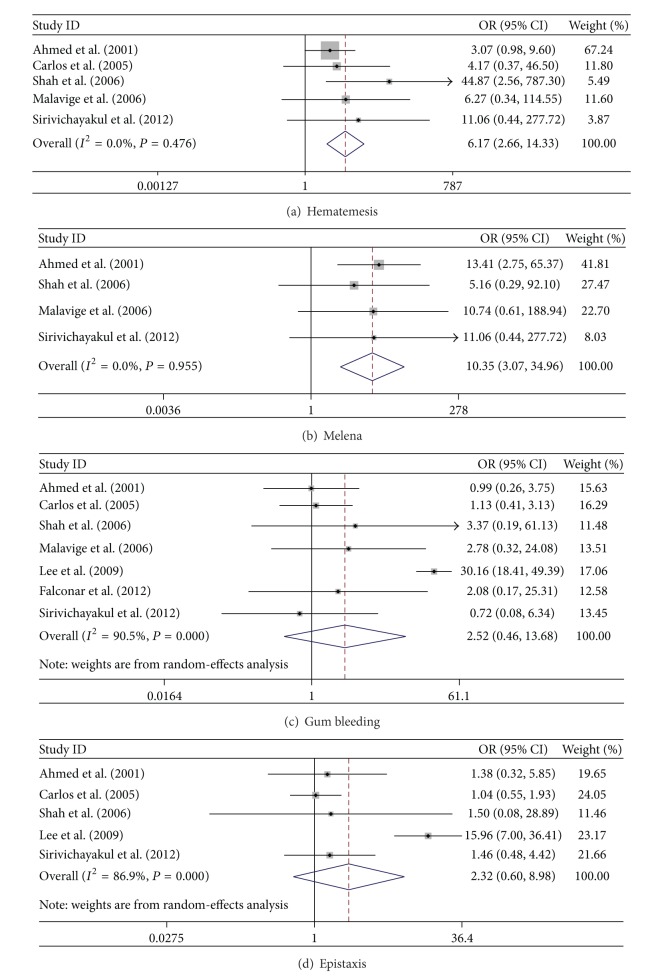
Forrest plots of four kinds of bleeding. (a)-(b) Pooled ORs of SDD are greater than one in hematemesis and melena; (c)-(d) pooled ORs of SDD are not significantly different from one in epistaxis and gum bleeding.

In this meta-analysis, the fix-effect model was analyzed in gender, hematemesis, melena, and headache, while the random-effect model was used in vomiting/nausea, abdominal pain, skin rashes, bleeding (gum bleeding and epistaxis), lethargy, retroorbital pain, diarrhea, hepatomegaly, and tourniquet. According to the meta-analysis results, there were no significant differences between the DF and SDD groups in association with the following factors (*P* > 0.05, Table 2): gender of patients, clinical symptoms/signs of lethargy, retroorbital, diarrhea, and tourniquet test. There were significant differences in association between the two groups with the following factors: symptoms/signs of vomiting/nausea, abdominal pain, skin rashes, bleeding, and hepatomegaly (*P* < 0.05, Figures 2(a)–2(e)). In particular, bleeding and hepatomegaly were highly correlated with the progression of DF into SDD, with the ORs at 13.617 (95% CI: 3.281, 56.508) and 4.751 (95% CI: 1.769, 12.570). The results indicate that these two factors strongly predict greater risk of the development of SDD. The other factors such as vomiting/nausea, abdominal pain, and skin rashes were also associated with SDD, while the strength of association was not as strong. In the bleeding symptoms, the ORs for predicting SDD of hematemesis and melena were 6.174 (95% CI: 2.66, 14.334; *P* < 0.001) and 10.351 (95% CI: 3.065, 34.956; *P* < 0.001), respectively, demonstrating significant differences between the DF and SDD groups, while the frequencies of the other two kinds of bleeding, gum bleeding and epistaxis, were not significantly different between the two groups. The details were shown in Table 2, Figures 2 and 3. Interestingly, headache was not associated with the low risk of SDD (OR: 0.555; 95% CI: 0.455, 0.676; Figure 2(f)).

### 3.3. Heterogeneity Analysis

Metaregression analysis was conducted to examine which factors could have brought heterogeneity across the studies, and 11 clinical symptoms/signs were analyzed, including vomiting/nausea, abdominal pain, skin rashes, bleeding (epistaxis and gum bleeding), lethargy, retroorbital pain, diarrhea, hepatomegaly, and tourniquet test. It turned out that two factors, study design and population, contributed to the heterogeneity in the studies of gum bleeding (*P* < 0.10) and epistaxis (*P* < 0.10). Based on the subgroup analyses, the epistaxis ratio was not significantly different between DF and SDD groups in children (*P* = 0.562), while the difference was significant in adults (*P* < 0.001, OR: 14.139, 95% CI: 6.622, 30.187). Similar results applied to the subgroup analysis of gum bleeding based on the retrospective and prospective studies (data not shown).

### 3.4. Publication Bias

Funnel plots showed no publication bias in the studies covering vomiting/nausea, abdominal pain, skin rashes, bleeding, or retroorbital pain (Figure S1 and Table 2). The *P* values of Egger's and Begg's tests also suggested that publication bias had little impact on the results. There were three signs, bleeding, gum bleeding, and hepatomegaly showing publication bias (Egger's test: *P* = 0.041, 0.058, 0.014).

## 4. Discussion

The present study is the meta-analysis to comprehensively evaluate the correlation of clinical symptoms/signs with the development of SDD in patients with DF. The results showed that a total of five symptoms/signs significantly predict dengue patients progressing into SDD: vomiting/nausea, abdominal pain, skin rashes, bleeding, and hepatomegaly. The other five factors were not associated with the disease progression, including tourniquet versus nontourniquet, female versus male patients, lethargy, retroorbital pain, and diarrhea. We found that patients with bleeding after DENV infection had approximately a 14-fold increased risk for progression into SDD (including DHF and DSS). When compared with the frequencies of leucopenia and thrombocytopenia, haemorrhagic manifestations, such as gum bleeding, epistaxis, and gastrointestinal bleeding, are less frequent, but not rare [28]. Our analysis included four kinds of bleeding: hematemesis, melena, gum bleeding, and epistaxis. Previous studies showed that the frequencies of hematemesis, melena, gum bleeding, and epistaxis were higher in SDD patients than in DF patients, but none of them were related to the risk of development of SDD in patients with these symptoms [1, 17, 18, 25]. A recent study also showed that the gastrointestinal bleeding was associated with DSS, although it is not a strong association (OR = 1.84) [29]. According to our meta-analysis, the two kinds of gastrointestinal bleeding that strongly predicted SDD were hematemesis (OR: 6.174; 95% CI: 2.66, 14.334; *P* < 0.001) and melena (OR: 10.351; 95% CI: 3.065, 34.956; *P* < 0.001), while the other two kinds of bleeding were not significant risk factors. The other four clinical symptoms and signs proved significant for predicting the progression into SDD are vomiting/nausea (OR: 1.692; 95% CI: 1.256, 2.280), abdominal pain (OR: 2.278; 95% CI: 1631, 3.182), skin rashes (OR: 2.031; 95% CI: 1.269, 3.250), and hepatomegaly (OR: 4.751; 95% CI: 1.769, 12.570). Although the vomiting/nausea, abdominal pain, and skin rashes showed a weak association with SDD compared with DF patients, these warnings must be taken seriously as recent studies demonstrated that these symptoms were associated with the mortality caused by dengue [30, 31]. We found that patients with hepatomegaly after DENV infection had approximately a 5-fold increased risk of progression into SDD; however, the CI was with a wide range. The possible reason is that the rate of hepatomegaly was significantly higher in adults than the elderly [32].

The most accepted hypothesis for progression of DF is that subneutralizing levels of DENV-specific antibodies exacerbate the disease by means of an antibody-dependent enhancement of infection (ADE) [33], which induces a complicated immunopathogenesis in the host. The extent of vascular permeability is enhanced as a result of ADE [34] and patients with SDD as well as alterations of endothelial cells have been shown to experience thrombocytopenia and coagulation disorders [35]. These significant symptoms/signs, especially the bleeding (hematemesis/melena) and hepatomegaly, are manifested in patients with SDD as a result of the aforementioned alterations. In the* in vivo* model for ADE-induced SDD, gastrointestinal bleeding and viral RNA increased in the liver were observed [36]. Additionally, based on skin biopsies, IgM, beta 1 C-globulin, dengue antigen, and fibrinogen deposits were found to be present within or about blood vessel walls of dermal papillae or in the blood vessels [37], implying that skin rashes that appeared in DHF were caused by an immunopathologic process. So in patients with SDD, the host immune system plays a central role in triggering symptoms like bleeding, hepatomegaly, and skin rashes, which could be used to triage patients in need of intensive care.

The unassociated factors/manifestations were gender, lethargy, retroorbital pain, diarrhea, and positivity of a tourniquet test. However, the World Health Organization (WHO) has published guidelines stating that positivity of a tourniquet test may be included in the clinical case definition of dengue haemorrhagic fever [38], and an altered level of consciousness such as lethargy should be paid extra attention [2]. Although the results from this meta-analysis showed unexpected absence of associations, relaxing vigilance over the patients with these symptoms/signs is not recommended, because the results were generated from a random-effect model that tends to be overconservative.

Furthermore, headache was a protective factor against SDD after DENV infection (OR: 0.555; 95% CI: 0.455, 0.676), implying that dengue patients with headache had a lower probability to develop into SDD. The protective effect has been proved by a retrospective cohort study [25]. However, in another study, BALB/c mice were infected with different strains of DENV which were isolated from DSS or DF patients, respectively, and in the mice infected with the strain from DSS patients, DENV-1 isolates appeared to be primarily neurotropic, whereas in the cases of other strains the virus turned to mainly infect lung and liver [39]. Suggesting that high frequency of headache could occur in patients with SDD.

There are limitations in the present study. Firstly, the results will not apply to multicenter prospective studies, since the present meta-analysis only included retrospective and single-center prospective studies. These designs could not eliminate recall and selection biases. Hence, the true associations between these symptoms/signs and the development of SDD might have been distorted. Secondly, the definitions of DF and SDD within these studies may have varied, which brought uncertainty into determining cases. Lastly, some of the results were based on a random-effect model that might weaken the validity of the analysis. Nonetheless, this study explored a new approach to identify the correlations of the symptoms/signs after DENV infection with the risk of progression into SDD, which can greatly facilitate the prevention of SDD.

## 5. Conclusions

This meta-analysis identified clinical symptoms and signs that significantly predicted DF patients progressing into severe dengue. DF patients with vomiting/nausea, abdominal pain, skin rashes, bleeding (hematemesis/melena), and hepatomegaly were more likely to develop SDD, while patients with headache had a lower risk of progression into SDD. Other factors such as gender, lethargy, retroorbital pain, diarrhea, and positive tourniquet test are not associated with SDD. Further studies, especially ones with larger sample sizes and prospective, are warranted to confirm the findings.

## Supplementary Material

Supporting information: PRIMSA checklist and publication bias in this meta-analysis.

## Figures and Tables

**Table 1:** Basic features of the eligible studies.

Author (publication year)	Study design	Population	Gender		Vomiting/nausea		Bleeding		Headache		Abdominal pain		Retroorbital pain		Rashes		Diarrhea		Hepatomegaly		Lethargy		Tourniquet test	
			DF (M/F)	SD (M/F)	DF (Y/N)	SD (Y/N)	DF (Y/N)	SD (Y/N)	DF (Y/N)	SD (Y/N)	DF (Y/N)	SD (Y/N)	DF (Y/N)	SD (Y/N)	DF (Y/N)	SD (Y/N)	DF (Y/N)	SD (Y/N)	DF (Y/N)	SD (Y/N)	DF (Y/N)	SD (Y/N)	DF (Y/N)	SD (Y/N)
Ahmed et al. (2001) [17]	Prospective	Children	—	—	4/22	5/41	16/10	46/0	22/4	34/12	—	—	7/19	10/36	3/23	24/22	6/20	8/38	0/26	36/10	—	—	—	—
Narayanan et al. (2002) [21]	Prospective	Children	22/21	9/7	34/9	15/1	23/20	16/0	13/30	4/12	10/33	4/12	3/40	4/12	4/39	1/15	—	—	19/24	12/4	11/32	3/13	5/38	9/7
Endy et al. (2002) [19]	Prospective	Children	—	—	44/89	14/5	2/130	1/18	82/50	14/5	20/111	6/13	—	—	6/125	1/18	5/127	1/18	—	—	43/89	6/13	—	—
Phuong et al. (2004) [22]	Prospective	Children	—	—	178/134	234/85	56/256	36/283	140/167	88/230	156/156	234/84	—	—	—	—	—	—	—	—	—	—	106/166	128/145
Carlos et al. (2005) [18]	Prospective	Children	143/96	72/48	—	—	11/221	6/105	—	—	69/168	51/68	—	—	—	—	—	—	—	—	0/238	4/115	—	—
Shah et al. (2006) [9]	Prospective	Children	—	—	11/0	87/2	0/11	68/21	11/0	87/2	9/2	87/2	11/0	87/2	—	—	—	—	0/11	77/12	11/0	87/2	10/1	15/15
Malavige et al. (2006) [23]	Prospective	Adult	—	—	18/15	51/24	5/28	37/38	26/7	45/30	3/30	14/61	—	—	—	—	7/26	24/51	10/23	39/36	—	—	—	—
Lee et al. (2006) [24]	Retrospective	Both	177/235	119/113	—	—	—	—	—	—	—	—	—	—	—	—	—	—	—	—	—	—	—	—
Riaz et al. (2009) [27]	Cross-sectional	Both	—	—	134/35	83/26	6/163	89/20	23/146	13/96	85/84	61/48	0/169	1/108	55/114	37/72	10/159	2/107	1/168	1/108	—	—	—	—
Lee et al. (2009) [25]	Retrospective	Adult	1183/672	53/29	604/1251	29/53	—	—	606/1249	16/66	252/1603	14/68	5/1850	0/82	887/968	49/33	—	—	—	—	—	—	—	—
Priyadarshini et al. (2010) [10]	Prospective	Both	—	—	90/69	44/18	—	—	101/58	33/29	29/130	31/31	34/125	2/60	31/128	26/36	—	—	—	—	—	—	—	—
Khan et al. (2010) [8]	Retrospective	Both	99/62	30/10	93/68	27/13	6/155	29/11	21/140	2/38	6/80	1/10	—	—	44/117	31/9	26/135	5/35	—	—	—	—	—	—
Giraldo et al. (2011) [7]	Retrospective	Children	77/74	16/14	96/55	23/7	51/100	14/16	92/59	17/13	78/73	22/8	18/133	4/26	63/88	16/14	29/122	5/25	27/124	7/23	54/97	20/10	—	—
Falconar et al. (2012) [20]	Prospective	Both	—	—	13/15	4/1	13/28	5/0	—	—	0/28	5/0	22/6	4/1	—	—	—	—	0/28	2/3	—	—	8/20	5/0
Karoli et al. (2012) [26]	Cross-sectional	Both	—	—	—	—	13/83	42/0	77/19	28/14	—	—	—	—	26/70	12/30	—	—	47/49	25/17	—	—	—	—
Sirivichayakul et al. (2012) [11]	Prospective	Children	—	—	95/28	32/2	—	—	107/16	25/9	59/64	25/9	35/88	13/21	53/70	18/16	21/102	17/17	—	—	23/100	6/28	—	—

Note: “M”: male; “F”: female; “Y”: yes, the group has the symptom; “N”: no, the group has no symptom. “—”: no statistics.

**Table 2:** Results of meta-analysis for the clinical manifestations between DF and SDD.

Clinical manifestation	Number of studies	Odds ratio (95% CI)	Test for OR	Test of heterogeneity		Publication bias	
			*P*	*I* ^2^ (%)	*P*	Egger's test	Begg's test
*Gender *	6	1.230 (0.999, 1.513)	0.051	0	0.686	0.991	0.707
Vomiting/nausea∗	13	1.692 (1.256, 2.280)	0.001	39.7	0.069	0.455	0.428
Abdominal pain∗	13	2.278 (1.631, 3.182)	<0.001	55.5	0.008	0.343	0.669
Skin rashes∗	10	2.031 (1.269, 3.250)	0.003	69.7	<0.001	0.581	0.592
Bleeding∗	12	13.617 (3.281, 56.508)	0.001	94.0	<0.001	0.033	0.373
*Hematemesis* ∗	5	6.174 (2.66, 14.334)	<0.001	0	0.476	0.137	0.211
*Melena* ∗	4	10.351 (3.065, 34.956)	<0.001	0	0.955	0.36	0.734
Gum bleeding	7	2.518 (0.463, 13.685)	0.285	90.5	<0.001	0.058	0.548
Epistaxis	5	2.319 (0.599, 8.976)	0.562	86.9	<0.001	0.981	0.462
*Headache* ∗	13	0.555 (0.455, 0.676)	<0.001	0	0.594	0.177	0.583
Lethargy	6	1.552 (0.714, 3.370)	0.267	49.6	0.078	0.864	1
Retroorbital pain	9	1.096 (0.531, 2.261)	0.804	45.2	0.067	0.810	0.602
Diarrhea	7	1.149 (0.555, 2.380)	0.708	64.1	0.010	0.185	0.230
Hepatomegaly∗	8	4.751 (1.769, 12.570)	0.002	72.5	0.001	0.014	0.063
Tourniquet	4	2.194 (0.395, 12.206)	0.369	82.4	0.001	0.715	1

Note: “Italics”: using the fixed-effect model, the other symptoms/signs: using the random-effect model; ∗significantly different between DF and SDD.

## Abbreviations

DENV: Dengue virus
DF: Dengue fever
SDD: Severe dengue disease
DHF: Dengue haemorrhagic fever
DSS: Dengue shock syndrome
NOS: The Newcastle-Ottawa Scale
ADE: Antibody-dependent enhancement of infection
WHO: The World Health Organization.

## References

[1] Malavige GN, Fernando S, Fernando DJ, Seneviratne SL (2004). Dengue viral infections. *Postgraduate Medical Journal*.

[2] Research SPF, Diseases TIT, Diseases WHOD, Epidemic WHO, Alert P (2009). *Dengue, Guidelines for Diagnosis, Treatment, Prevention and Control*.

[3] Chevillon C, Failloux A-B (2003). Questions on viral population biology to complete dengue puzzle. *Trends in Microbiology*.

[4] Rigau-Pérez JG, Clark GG, Gubler DJ, Reiter P, Sanders EJ, Vorndam AV (1998). Dengue and dengue haemorrhagic fever. *Lancet*.

[5] http://www.who.int/csr/disease/dengue/impact/en/.

[6] Khan E, Siddiqui J, Shakoor S, Mehraj V, Jamil B, Hasan R (2007). Dengue outbreak in Karachi, Pakistan, 2006: experience at a tertiary care center. *Transactions of the Royal Society of Tropical Medicine and Hygiene*.

[7] Giraldo D, Sant'Anna C, Périssé AR (2011). Characteristics of children hospitalized with dengue fever in an outbreak in Rio de Janeiro, Brazil. *Transactions of the Royal Society of Tropical Medicine and Hygiene*.

[8] Khan E, Kisat M, Khan N, Nasir A, Ayub S, Hasan R (2010). Demographic and clinical features of dengue fever in Pakistan from 2003-2007: A retrospective cross- sectional study. *PLoS ONE*.

[9] Shah GS, Islam S, Das BK (2006). Clinical and laboratory profile of dengue infection in children. *Kathmandu University Medical Journal*.

[10] Priyadarshini D, Gadia RR, Tripathy A (2010). Clinical findings and pro-inflammatory cytokines in dengue patients in Western India: a facility-based study. *PLoS ONE*.

[11] Sirivichayakul C, Limkittikul K, Chanthavanich P (2012). Dengue infection in children in ratchaburi, thailand: a cohort study II. clinical manifestations. *PLoS Neglected Tropical Diseases*.

[12] Pawitan JA (2011). Dengue virus infection: predictors for severe dengue.. *Acta Medica Indonesiana*.

[13] Lima FR, Croda MG, Muniz DA, Gomes IT, Soares KR, Cardoso MR (2013). Evaluation of the traditional and revised World Health Organization classifications of dengue cases in Brazil. *Clinics*.

[14] Moher D, Liberati A, Tetzlaff J, Altman DG (2009). Preferred reporting items for systematic reviews and meta-analyses: the PRISMA statement. *PLoS Medicine*.

[15] Wells G, Shea B, Connell D O, Peterson J, Welch V The Newcastle-Ottawa Scale (NOS) for assessing the quality of nonrandomised studies in meta-analyses.

[16] Higgins JPT, Thompson SG, Deeks JJ, Altman DG (2003). Measuring inconsistency in meta-analyses. *British Medical Journal*.

[17] Ahmed FU, Mahmood CB, Sharma JD, Hoque SM, Zaman R, Hasan MS (2001). Dengue and dengue haemorrhagic fever in children during the 2000 outbreak in Chittagong, Bangladesh. *Dengue Bulletin*.

[18] Carlos CC, Oishi K, Cinco MTDD (2005). Comparison of clinical features and hematologic abnormalities between dengue fever and dengue hemorrhagic fever among children in the Philippines. *The American Journal of Tropical Medicine and Hygiene*.

[19] Endy TP, Chunsuttiwat S, Nisalak A (2002). Epidemiology of inapparent and symptomatic acute dengue virus infection: a prospective study of primary school children in Kamphaeng Phet, Thailand. *American Journal of Epidemiology*.

[20] Falconar CM, Falconar AK, Romero-Vivas CM (2012). Simple prognostic criteria can definitively identify patients who develop severe versus non-severe Dengue disease, or have other febrile illnesses. *Journal of Clinical Medicine Research*.

[21] Narayanan M, Aravind MA, Thilothammal N, Prema R, Sargunam CSR, Ramamurty N (2002). Dengue fever epidemic in Chennai—a study of clinical profile and outcome. *Indian Pediatrics*.

[22] Phuong CXT, Nhan NT, Kneen R (2004). Clinical diagnosis and assessment of severity of confirmed dengue infections in Vietnamese children: is the world health organization classification system helpful?. *The American Journal of Tropical Medicine and Hygiene*.

[23] Malavige GN, Velathanthiri VGNS, Wijewickrama ES (2006). Patterns of disease among adults hospitalized with dengue infections. *QJM*.

[24] Lee MS, Hwang KP, Chen TC, Lu PL, Chen TP (2006). Clinical characteristics of dengue and dengue hemorrhagic fever in a medical center of southern Taiwan during the 2002 epidemic. *Journal of Microbiology, Immunology and Infection*.

[25] Lee VJ, Lye DC, Sun Y, Leo YS (2009). Decision tree algorithm in deciding hospitalization for adult patients with dengue haemorrhagic fever in Singapore. *Tropical Medicine and International Health*.

[26] Karoli R, Fatima J, Siddiqi Z, Kazmi KI, Sultania AR (2012). Clinical profile of dengue infection at a teaching hospital in North India. *The Journal of Infection in Developing Countries*.

[27] Riaz MM, Mumtaz K, Khan MS (2009). Outbreak of dengue fever in Karachi 2006: a clinical perspective. *Journal of the Pakistan Medical Association*.

[28] Rigau-Pérez JG, Clark GG, Gubler DJ, Reiter P, Sanders EJ, Vorndam AV (1998). Dengue and dengue haemorrhagic fever. *The Lancet*.

[29] Huy NT, van Giang T, Thuy DH (2013). Factors associated with dengue shock syndrome: a systematic review and meta-analysis. *PLOS Neglected Tropical Diseases*.

[30] Mena LA, Fernandez J, Morales A, Soto Y, Feris-Iglesias J, Brito MO (2014). Disease severity and mortality caused by dengue in a Dominican pediatric population. *The American Journal of Tropical Medicine and Hygiene*.

[31] Thein TL, Leo YS, Fisher DA (2013). Risk factors for fatality among confirmed adult dengue inpatients in Singapore: a matched case-control study. *PLoS ONE*.

[32] Rowe EK, Leo YS, Wong JG (2014). Challenges in dengue fever in the elderly: atypical presentation and risk of severe dengue and hospita-acquired infection. *PLoS Neglected Tropical Diseases*.

[33] Halstead SB (2003). Neutralization and antibody-dependent enhancement of dengue viruses. *Advances in Virus Research*.

[34] Halstead SB, Mahalingam S, Marovich MA, Ubol S, Mosser DM (2010). Intrinsic antibody-dependent enhancement of microbial infection in macrophages: disease regulation by immune complexes. *The Lancet Infectious Diseases*.

[35] Krishnamurti C, Kalayanarooj S, Cutting MA (2001). Mechanisms of hemorrhage in dengue without circulatory collapse. *The American Journal of Tropical Medicine and Hygiene*.

[36] Zellweger RM, Prestwood TR, Shresta S (2010). Enhanced infection of liver sinusoidal endothelial cells in a mouse model of antibody-induced severe dengue disease. *Cell Host and Microbe*.

[37] Boonpucknavig S, Boonpucknavig V, Bhamarapravati N, Nimmannitya S (1979). Immunofluorescence study of skin rash in patients with dengue hemorrhagic fever. *Archives of Pathology and Laboratory Medicine*.

[38] WHO (1997). *Dengue Haemorrhagic Fever: Diagnosis, Treatment, Prevention and Control*.

[39] Tuiskunen A, Wahlström M, Bergström J, Buchy P, Leparc-Goffart I, Lundkvist Å (2011). Phenotypic characterization of patient dengue virus isolates in BALB/c mice differentiates dengue fever and dengue hemorrhagic fever from dengue shock syndrome. *Virology Journal*.

